# Application of an Adapted Behaviour Change Wheel to Assistance Dog Puppy Raising: A Proposed Raiser-Centred Support Program

**DOI:** 10.3390/ani13020307

**Published:** 2023-01-16

**Authors:** Dac L. Mai, Tiffani Howell, Pree Benton, Pauleen C. Bennett

**Affiliations:** 1 Anthrozoology Research Group, Department of Psychology, Counselling and Therapy, School of Psychology and Public Health, La Trobe University, Flora Hill, VIC 3552, Australia; 2 Centre for Service and Therapy Dogs Australia, Melbourne, VIC 3162, Australia

**Keywords:** organisational support, social support, puppy foster carer, behaviour change wheel, puppy raising model

## Abstract

**Simple Summary:**

Puppy raisers volunteer their time to take care of, and train, selected puppies until they are mature enough to undertake advanced training to become assistance dogs. Emerging evidence suggests a central role for puppy raisers in bringing out the best in assistant dog puppies. However, most puppy raisers are not professional dog trainers and, to optimise outcomes, they may require various kinds of support from the host organisation that places the puppy in their home, as well as from their personal networks and local communities. From a behaviour change perspective, coordinated efforts at different levels would be expected to better enable puppy raisers to improve training outcomes for the puppies. The aim of this paper is to discuss the potential application of a well-established behaviour change framework to the practice of puppy raising. After discussing relevant findings, we suggest a holistic approach to supporting puppy raisers and propose a behaviour model to help volunteers improve their puppy raising practice.

**Abstract:**

Puppy raising (PR) programs recruit volunteer community members (raisers) to raise assistance dog (AD) candidates from puppyhood until the dogs are ready for advanced training. Once qualified, ADs assist human handlers with a disability to live more independently. Unfortunately, about 50% of all puppies do not meet the behavioural standards required for further training after completing a PR program. This increases costs and lengthens the time taken for a handler to obtain an AD. Research has identified several factors that influence raisers’ experiences. It has also shown that raisers’ socialisation and training practices affect perceived puppy behaviour. Drawing on the argument that puppy raiser practices are central to improving overall puppy raising program outcomes, this paper interprets recent findings within the framework provided by the behaviour change wheel—an established behaviour change framework—to suggest a coordinated approach to supporting puppy raisers. The recommendations will allow future research to employ more objective measures and more rigorous experimental designs as the field attempts to corroborate existing findings and develop evidence-based models of practice.

## 1. Introduction

High demand for assistance dogs (ADs) has emphasised the importance of appropriate selection and training of AD candidates, to increase their chance of meeting high performance and behaviour standards. Many potential ADs are selected as puppies, following which they typically live with volunteer raisers during a puppy raising (PR) program that may last for up to a year. During such programs the puppy learns life skills and basic education, enabling them to proceed to advanced training if suitable [1,2]. Efforts made to improve PR outcomes have focused on enrolling high-quality puppies [3,4] and examining different program designs [5]. However, PR programs often report high failure rates, with approximately 40% of all puppies eliminated at the end of a PR program in one study [6], and up to 50% of all guide dog puppies in other studies failing to pass their final assessment to qualify as guide dogs [7,8].

Puppy behaviour is influenced by genetics and environmental factors during all stages of development: prenatal, neonatal, transition, socialisation, juvenile, and pubertal periods [9]. Experienced breeders and AD organisations (e.g., [6]) might be expected to have access to the expertise and resources required to optimise the development of puppy behaviour during the first three of these stages. However, the last three developmental stages are imperative to consolidate desirable traits and develop new adaptive behaviours [9,10,11,12]. These three stages, occurring between 8 weeks to 16 months of age, take place when puppies are typically being cared for by a volunteer puppy raiser. Indeed, some AD puppies may transition through multiple homes, a practice less than ideal from an ethical or practical perspective given the demonstrated existence of sensitive socialisation periods. Raising AD puppies to high standards is a demanding and prolonged undertaking, requiring a sustained level of commitment by dedicated and effective puppy raisers. We contend, therefore, that efforts to improve overall PR program outcomes should aim, first, to improve raiser practices and, second, to retain successful raisers. 

These objectives are consistent with a behaviour change approach in which contextual factors such as organisational support can encourage desirable behaviours (i.e., what puppy raisers do to help their puppies) and discourage undesirable behaviours (i.e., what raisers do that may hinder the puppies’ training outcomes). The aim of this paper is to discuss the potential application of this behaviour change framework to PR practices and research in this field. This paper firstly summarises findings from three recent studies. These studies were concerned with raiser practices and their influences on puppies’ behaviours and included a cross-sectional qualitative study [13], an online survey study [14], and a longitudinal qualitative study [15]. A key finding is that raisers are central to the overall outcomes of a PR program. Therefore, the paper then introduces a behavioural model (i.e., the behaviour change wheel [16]) that could be applied to supporting puppy raisers. The expected outcomes of this approach include the improvement of raisers’ practices and, consequently, puppies’ behavioural development and training progress, as well as increased retention of successful puppy raisers. 

## 2. Human Factors That Influence Successful Puppy Raising

### 2.1. Predictors of Puppy Behaviour during a PR Program, as Perceived by Puppy Raisers

Because puppy behaviour is a predominant outcome variable in current AD research, two studies, a cross-sectional qualitative study [13] and an online survey study [14], were designed to explore predictors of perceived puppy behaviour. The qualitative study [13] recruited 17 participants, comprising experienced raisers and/or staff from different AD programs. The raisers were asked about their PR experiences and staff were asked what they thought about the experiences of raisers. In the online survey study [14], 231 puppy raisers (205 women, 25 men, one undisclosed; aged between 18 and 79 years old) from seven countries (mostly the United States) completed an online survey. The survey collected raisers’ demographic details, ratings of their puppies’ behaviour, information about their raising practices, and the perceived helpfulness of various sources of support. Both studies looked beyond puppy-specific factors (e.g., puppies’ breed, sex, age, and baseline characteristics that they develop before joining the PR program) and considered raiser-specific and contextual factors as candidate predictors of puppies’ behaviour.

The initial study [13] revealed several factors perceived by participants as being helpful or unhelpful to the PR process. Those factors included raisers’ personal differences (i.e., expectations, competency, help-seeking behaviours, perseverance, and passion); informational and emotional support from program providers and those in the raisers’ personal networks and puppy characteristics, such as age and breed. These themes were used to inform the development of an online survey, which was used to quantitatively examine how the various factors related to each other and to how raisers rated their puppies’ behaviour. The second study [14] showed that puppy breed, sex, and intended future AD role were not significantly related to perceived puppy behaviours, with puppy age being the only puppy-specific factor that was statistically relevant to be included in further analyses. After controlling for puppy age, raiser socialisation and training practices, the variables that consistently appeared in models predicting most of the puppies’ perceived behaviours were: trainability, general anxiety, adaptability, excitability, and distractibility. 

A limitation of the survey study is that it was not possible to fully characterise what the respondents meant by the words socialisation or training–socialisation, for example, which can be used to refer to age-appropriate exposure to various stimuli under controlled conditions or to uncontrolled overexposure of puppies to new and frightening stimuli which may inadvertently cause anxiety and stress [11]. However, attempts were made to improve the sensitivity of items in the Raiser Practices Questionnaire [14] by referring to ‘sufficient’ training and exposure to a variety of social and non-social stimuli in ‘a safe and controlled manner’ (i.e., “I put enough time into my puppy’s socialisation”; “I ensure that my puppy is only exposed to new experiences in a safe and positive way”). Although the participants in these two studies [13,14] came from different PR programs with varying degrees of standard training protocols and typically requiring no prior training to volunteer for the role, together, the findings from these two studies [13,14] suggest that raisers’ practices may be among the best predictors of puppies’ behaviour.

The only significant organisational support predictor in the online survey study [14] was the availability of puppy sitters, people who helped raisers take care of their puppies on a short-term basis (e.g., for a few hours during a busy day or on long weekends). Support from these temporary sources predicted lower raiser ratings of puppy excitability. Both studies [13,14] also identified several other individuals who shared puppy raising responsibilities with raisers. These included family members, friends, and colleagues. This finding was in line with Chur-Hansen et al. [17] regarding the involvement of such individuals in PR activities. It is possible that support practices (i.e., socialisation and training) from puppy sitters enabled raisers to influence puppies’ behaviour directly. It could also be that for raisers who received more support from puppy sitters, their puppies had more opportunity to socialise with a wider range of people and became less excitable, which was the only puppy behaviour that was affected by this type of support.

Although access to puppy sitters influenced puppies’ perceived excitability, it is instructive that raisers with higher ratings on their socialisation and training practices rated not only their puppy’s excitability more favourably, but also their trainability, general anxiety, adaptability, and distractibility. It was therefore concluded that raisers’ socialisation and training practices may be among the best predictors of puppies’ behaviour and that, consequently, understanding and promoting factors that contribute to puppy raisers’ engagement in optimal training and socialisation practices is an important area for future research and practice in this field. 

### 2.2. Facilitators and Barriers to Raisers’ Engagement in Recommended PR Practices

To understand the nature of, and factors that affect, raisers’ practices, a longitudinal qualitative study [15] was conducted in parallel with the abovementioned cross-sectional ones [13,14]. This study [15] closely followed eight puppy raisers who were participating in a PR program based at a university campus in a regional city of Australia. An AD partner organisation was responsible for all aspects of this program, such as screening and selecting prospective raisers from the university community, puppy placements, weekly training, and meeting all expenses associated with raising the puppies. Interviews with the raisers took place monthly, from before they received their puppy until they completed the program. Sixteen interviews were pseudo-randomly selected for analysis, revealing several facilitators and barriers to the raisers’ engagement in frequent socialisation, consistent training, and effective ongoing learning. 

Foremost amongst the identified predictors of raisers’ practices was the provision of support from the AD organisation that supplied the puppies, which provided both informational and emotional benefits to raisers. Support from other sources was also identified as important. Although the survey findings [14] showed that only support from experienced raisers (mentors) from the same PR program predicted any statistical improvement in the raisers’ socialisation and training practices, the longitudinal qualitative study [15] suggested that dog trainers not from the PR program, as well as co-workers, family members, and friends, provided raisers with emotional support. This emotional support was critical in terms of ensuring that the experience of puppy raising was positive, an outcome variable not measured in the survey study [14] but likely to be important in terms of retaining experienced volunteers. It was also found in the survey study that mentors were important not only in terms of effectively delivering organisation-specific instructions to the raisers but also because they promoted raisers’ help-seeking behaviour. Raisers who sought help rated more highly on puppy socialisation and training practices, confirming that raisers should be encouraged to be active recipients of support and to determine the type and amount of support needed to benefit their PR practices.

An additional finding of note from the longitudinal study was an interaction between puppy characteristics and raiser practices. There were clear benefits associated with having an easy-going puppy and several negative impacts of raising a high-energy puppy. Raisers in the survey study [13] associated positive experiences with laid-back puppy temperaments, and the longitudinal findings [15] explained that some raisers were reluctant to accompany distracted and high energy puppies in public places, which then reduced socialisation opportunities for those puppies. Raisers’ perception of their puppy as a nuisance was reported as a reason for negative PR experiences in past research [17]. It may be that puppies that are easy to handle receive sufficient opportunities to train and socialise in various public settings, which further benefits their training program. Due to the non-experimental nature of the studies being reviewed in this commentary, however, causal inferences cannot be supported.

### 2.3. Towards a Raiser-Centred Support Programs

The influences of contextual and puppy factors on raiser practices can be interpreted using frameworks developed in family-centred research in human development. In a child developmental study, Kelly and Coughlan [18] interviewed parents about facilitators and barriers to their child’s recovery from mental health issues. In this study, parents, as key stakeholders and joint decision-makers on mental health service use for their child, reported that their efforts and their relationship with their child facilitated the child’s recovery. Several benefits from professional mental health services included provision of information regarding the child’s mental health condition, and communication and rapport amongst professionals, parents, and children. Other contextual factors included messages from the media and levels of understanding from the child’s school. Professionals and other contextual factors were revealed as both facilitators and barriers depending on the nature of their interactions with both the parents and their children.

The parents in Kelly and Coughlan’s study [18] played a crucial role in determining the type and amount of mental health services accessed and they regulated the impacts of various contextual factors on their child’s mental health recovery. This role appears analogous to PR practices, whereby raisers (akin to parents) help improve their puppies’ behaviour by complying with instructions from the administering organisation and accessing various contextual forms of support (akin to mental health services). This resemblance between the influences of parents on children and raisers on AD puppies suggests that AD associations could benefit from evidence emerging from research in parenting practices. Similar to a parent-centred approach in child development research e.g., [18,19], PR research should focus on raiser-centred support programs that enable raisers to improve their practices and their puppies’ behaviour. 

We could not find any studies in the AD training literature that examine those programs from a raiser- or owner-centred approach. Indeed, a focus on the experiences of raisers and on when and how they apply different strategies is absent from the available literature. This is important because most dog training programs are aimed at providing information directly to PR raisers or companion dog owners (i.e., instructing them regarding how to handle their puppies). This reflects an assumption that efforts to better inform raisers’ practices will benefit their puppies’ behaviour. This assumption may not be warranted, however, because provision of information may not be sufficient to engender behaviour change. Instead, the relationship between puppy and raiser may be bidirectional, whereby puppy behaviour may benefit from and also affect the raiser’s PR practices. Such ‘child effects’ are well established in human family research [20,21,22]. 

## 3. A Raiser-Centred Behavioural Approach to Successful Puppy Raising

The transactional family dynamics theory [23] proposes that family members, via mutual relationships, exert influences on each other (i.e., dyad) and on relationships between other family members (i.e., family-wide). At the same time, they are affected by those dyad and family-wide relationships [22]. The child effects from the transactional family dynamics model [20,21,22], for instance, confirm that there are bidirectional relationships between children and caregivers. These transactional processes suggest that, not only do children passively receive parenting, but their presence and interaction patterns also influence their relationships with parents and/or the relationship between their parents [22,24]. Similar interaction patterns were reported by raisers in Chur-Hansen et al. [17], with AD puppies affecting raisers’ lives and family dynamics. For instance, undesirable puppy behaviour negatively affected raisers’ relationships with family members and those in their personal networks. Puppies’ disruptive behaviours and the endorsement of different puppy handling methods were reported to trigger disputes between the raisers and other family members, friends and members of the public, both in the first week of the program and again at three months, with fewer benefits reported at this time [17]. AD puppies may also form different relationships with different family members, resulting in marked differences in the perceived costs relative to the benefits of raising the puppy. When viewed from a family systems perspective (e.g., parent–child interactions) [22,23], these raiser–puppy dynamics suggest a potential for utilising behavioural support programs to change unhealthy patterns that may disadvantage the puppy’s training progress. 

Behavioural intervention refers to a process by which a set of actions are performed to promote desirable behaviour, or to reduce the likelihood of a specific undesirable behaviour, either by discouraging it or promoting engagement in an incompatible behaviour [16]. Although puppy behaviour remains an essential component of a PR program, in the following discussion the focus is on behaviour change at the raiser level. This reflects our evidence-based contention that promoting optimal PR patterns will improve raisers’ program experiences and, consequently, puppies’ training progress. Below, we introduce a robust behaviour framework called the behaviour change wheel (BCW) [16], which has guided successful development of behavioural interventions for human parents through parenting intervention programs e.g., [25,26,27,28], family-centred support programs for individuals with a disability [29] and peer-support programs to promote mothers’ breastfeeding practices [30]. We then adapt the BCW framework to develop a model for raiser-centred behaviour support programs aimed at promoting successful PR practices.

### 3.1. The Behaviour Change Wheel

**Figure 1:**
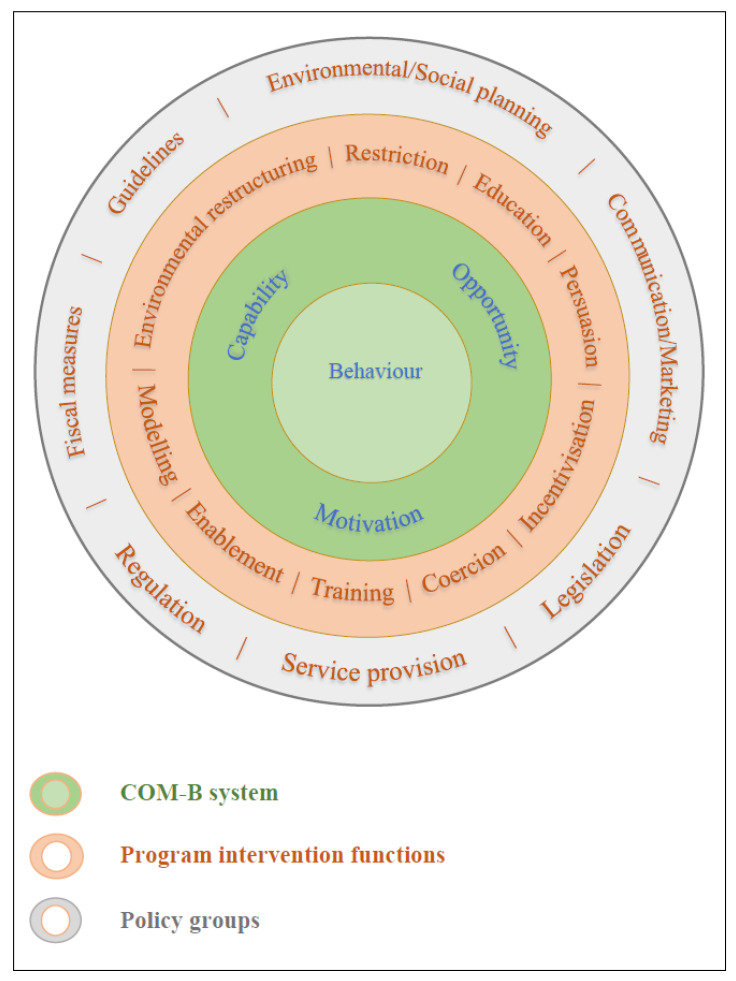
The COM-B System and the BCW Model, adapted from Michie et al. [16].

The behaviour change wheel (BCW) was synthesised from 19 behaviour theories, models, and frameworks [16] (see Figure 1). At its core, the ‘COM-B’ system proposes three conditions for behavioural changes to occur: capability, opportunity, and motivation. For an individual to perform a target behaviour (B), they need to have the capability (C) to perform it, the right opportunity (O), and motivation (M) to carry out that behaviour. For instance, puppy raisers bringing their puppies to a shopping mall for socialisation is a behaviour (B) that requires the knowledge and physical capability to perform the task effectively (C), time and access to a shopping mall (O), and the motivation to socialise the puppy in the shopping mall (M). The middle layer of the BCW comprises nine functions of behaviour intervention programs linked to the capability, opportunity, and motivation conditions of the COM-B system [16]. There are also seven policy groups in the outermost layer, relating to the broader societal context.

Definitions of the nine intervention functions are presented in Table 1, together with specific puppy-raising examples.

A comprehensive behaviour support program may aim to achieve all nine functions depending on the circumstances of the specific raiser-puppy team, but each support plan should be individualised to suit the raiser’s circumstances and their puppy’s training requirements. For instance, while focusing on providing training to puppy raisers (e.g., analytical skills and specific dog handling techniques), program providers may also restrict access to local areas with known aggressive dogs (i.e., restriction). The PR organisation may also collaborate with each raisers’ employer to accommodate their puppy at the workplace (i.e., environmental restructuring) and offer extra assistance from competent dog sitters who could provide supplementary or special socialisation and training sessions for puppies when raisers are not available (i.e., enablement). For some raisers, restrictions concerning contact with aggressive dogs may not be relevant in their local settings. Instead, they may need restrictions around approaching small animals to avoid developing undesirable behaviours such as pulling on the leash, barking, and/or chasing. As raisers and program providers agree on specific goals for each puppy (e.g., increasing socialisation for timid puppies), they may consider a behavioural intervention program to ensure raisers can achieve these goals. 

The outermost layer of the BCW model lists seven policy categories—the highest level of influence where peak bodies and governing authorities regulate their member organisations to promote or reduce certain behaviours at the individual level. Since the evidence discussed in this paper is limited to how organisations may help raisers to engage in optimal practices, the seven policy categories are beyond the scope of this commentary. Future research is needed to suggest the relevance of industry-level recommendations.

### 3.2. The Triple Triangle (3T) Model—A Holistic Approach to Improving Raisers’ PR Practices

**Figure 2:**
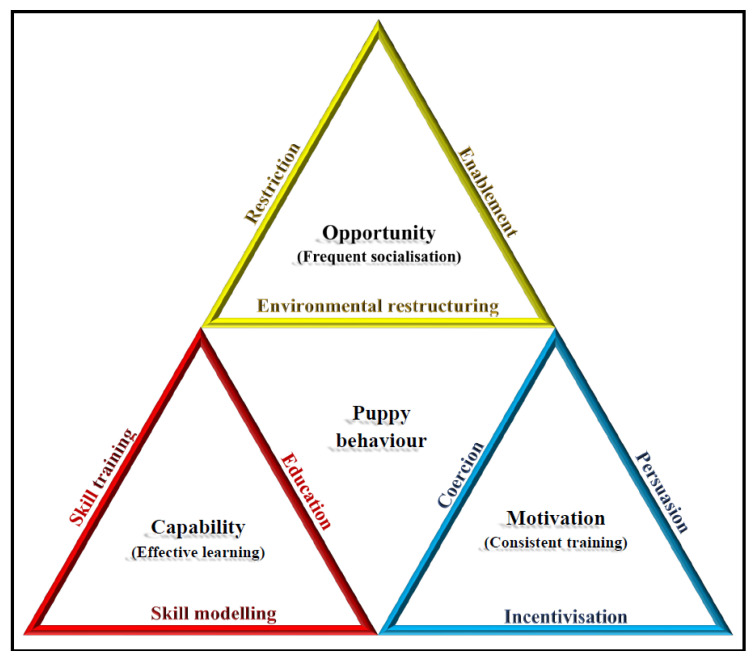
The Triple Triangle (3T) Model for Assistance Dog Puppy Raising.

As discussed above, the COM-B model and the nine intervention functions of the BCW framework provide a general approach for promoting an individual’s engagement in target behaviours, which can easily be applied to the PR context. This section extends this framework, proposing a raiser-centred behavioural support framework, called the triple triangle (3T) model (see Figure 2), which was adapted from the BCW’s individual- and program-level concepts to be specific to the findings from the mixed-method research [13,14,15] discussed earlier in this paper.

As the name suggests, the model encompasses three individual triangles representing the capability, opportunity, and motivation conditions in the COM-B system. The three triangles form a larger overarching triangle representing the holistic approach to improving raisers’ practices. The edges of the three triangles refer to the program functions to promote the COM-B condition that the respective triangle represents. For instance, to provide suitable opportunities for raisers to engage in a recommended behaviour, program providers may consider strategies relevant to environmental restructuring, enablement, and/or restriction functions. All nine functions are represented by the three triangles but, in the 3T model, each component triangle also targets one of three raiser practices that we believe are fundamental to effective puppy raising: provision of frequent socialisation and consistent training for the puppy, and effective learning (professional development) on the part of the raiser. 

Although the importance of promoting puppy socialisation and training is self-evident, providing raisers with ongoing training and professional development is equally necessary to ensure they can conduct effective puppy-focused practices (i.e., puppy socialisation and puppy training). Successfully performing those tasks requires appropriate education, skills training, and extensive skills modelling—the three edges of the capability triangle. This can sometimes be accomplished quite easily. Our longitudinal qualitative study [15], for example, showed that having training instructions available in various modalities was helpful to suit raisers’ preferred learning styles. The skills modelling function was also repeatedly supported by our findings, in which raisers preferred receiving support from experienced puppy raisers [13,14] and accrued benefits from learning from other puppy raisers [15]. In brief, when aiming to enhance raisers’ capability, their learning should be considered as the direct target behaviour.

The opportunity triangle is concerned with raisers’ puppy socialisation practices. Providing puppies with more socialisation appears to rely mostly on raisers having opportunities to perform relevant tasks. PR programs may consider strategies focusing on environmental restructuring, enablement, and/or restrictions to increase socialisation opportunities—the three edges of the opportunity triangle. As revealed in the longitudinal qualitative study [15], raisers reported that their puppy received more socialisation when their workplace had policies and equipment in place so they could bring the puppy to work (environmental restructuring). They also benefitted from having supplementary support from other family members, colleagues, and trained puppy socialisers, reflecting the enablement function of the opportunity triangle.

The last triangle is motivation, which, in the PR context, primarily refers to raisers’ engagement in consistent puppy training. Assuming that raisers have the necessary skills and competency and opportunities to facilitate frequent socialisation and training sessions with their puppy, the remaining condition is that raisers have the motivation to perform consistent and correct dog training techniques during these activities. This motivation can be enhanced if raisers perceive sufficient incentivisation, persuasion, or coercion associated with the tasks at hand. In Chur-Hansen et al.’s [17] study, raisers reported that producing successful puppies for future handlers and opportunities to enhance their families’ dynamics were motivations to join the PR program and to help with the puppies’ learning. These motivations should be harnessed to improve performance. In contrast, motivation to engage in behaviours contrary to organisation-specific training rules should be discouraged. Participants in one study [13] revealed that, in programs where raisers are permitted to keep unsuccessful puppies, raisers reluctant to return a puppy might be tempted to purposely cause it to fail. Disallowing retention of unsuccessful puppies could be used to de-motivate this behaviour.

Although the coercion function aims primarily to prevent undesirable practices, program providers should be aware of external factors that may prevent raisers from performing instructed tasks. For example, raisers in our study [15] reported perceiving negative judgements of some training techniques by members of the public, which reduced their motivation to engage in puppy training in public places. A similar sentiment was also reported in Chur-Hansen et al.’s [17] study regarding contradicting public opinions on the training regime. This finding implies coercion effects on raisers performing consistent puppy training, whereby perceived judgement from others becomes a punishment for adhering to the organisation’s recommended practices. Justification for recommended training practices should be included in professional development opportunities for raisers provided by program providers.

In short, the 3T model presents three triangles representing the three COM-B conditions to promote behaviour change, namely, capability, opportunity, and motivation. In general, puppy raising programs should ‘motivate’ raisers to carry out training that they have already been taught how to conduct (capability) and provide the right conditions (opportunity) for these behaviours to occur. Each condition targets a specific practice, namely, effective learning on the part of the puppy raiser, and frequent socialisation and consistent training for the puppy. The three edges of each triangle represent the program functions required to enhance each condition that the triangle represents. The larger triangle refers to the interconnectedness of different PR practices and their underlying behavioural principles. Central to the model are puppy characteristics, with this emphasising the ultimate purpose of the PR program—to produce an adult dog with desirable behaviour and temperament, who is healthy and who can perform the tasks relevant to their future assistance role.

## 4. Practical Implications and Recommendations

PR programs typically involve several stakeholders: raisers and their household members, friends and colleagues; members of the public; mentors and supporters; and staff from the host organisation. The practical implications of this paper correspond to those stakeholders, with clear recommendations emerging from the findings.

Prospective raisers should seek clarification of what is required of them and consider the skills and resources they have, and available organisational support, when deciding whether to become a puppy raiser. Interested volunteers may initially perceive many potential benefits. However, raising a successful puppy requires a long-term commitment and efforts that may be beyond what was originally anticipated. It may be instructive for potential raisers to gain experience through undertaking short-term fostering. Once they have joined a PR program, they should be encouraged to actively seek advice and assistance from staff and those with experience at their organisation. Ongoing professional development (learning) equips raisers with appropriate knowledge and skills. It subsequently helps puppies when raisers learn to arrange convenient and enjoyable opportunities for socialisation and when they persevere and are consistent with organisational requirements regarding training practices.

All household members should join prospective raisers in the decision-making process to raise a puppy. They should be aware of their influence on both the raiser and the puppy. During the PR program, household members are likely to be in close contact with, and involved in the training of, the puppy. Simply understanding and being respectful of the training process is essential. With skills, knowledge, and active involvement in the program, household members may share raising responsibilities and help raisers fulfil their commitment. Although friends and colleagues may be less involved than household members, it is important that they show understanding for the raiser’s volunteering commitment and perhaps assist when raisers bring a puppy to social events or workplaces. Friends, colleagues, and the raiser should establish boundaries to minimise any negative impacts on themselves, the puppy, and their social and workplace dynamics.

Members of the public may influence PR in many ways, ranging from enjoying the presence of AD puppies and appreciating raisers’ volunteer commitment through to expressing inappropriate personal opinions about contested training practices. Although social interactions may often be welcomed by raisers and helpful to puppies’ socialisation, unsolicited encounters may over-excite friendly puppies, frighten timid ones, and take up raiser time when they are busy or in a hurry. Therefore, it is recommended that members of the public always ask permission from raisers prior to any engagement. Furthermore, although public concerns and constructive feedback are essential to the sustainability of working dog industries [31], members of the public who disagree with how a puppy is being trained, perhaps due to beliefs about the perceived effectiveness of different training techniques, should be discouraged from approaching volunteer raisers, who are expected to execute the training program as instructed. Public concerns should, instead, be directed to responsible staff at the program provider, unless, of course, a puppy’s welfare is genuinely compromised and a threat is imminent. Beginning a conversation about a potentially controversial topic (e.g., training styles) with a raiser may counter the efforts and motivations of these volunteers. Conversely, experienced raisers and supporters (e.g., sitters and socialisers) should be aware of the positive impact they can have on other puppy raisers, especially novices. They should be encouraged to promote raisers’ help-seeking behaviours, provide their peers with technical and emotional support and have more active involvement. Through doing this, they can contribute substantially to the improvement of puppies’ behavioural outcomes.

Staff responsible for raiser training and support should consider several aspects of each raiser’s life, as various factors may counter both the raiser’s and the organisation’s efforts. Administratively, program providers should assess risks and hazards associated with raisers’ fitness, living environments, and local and work settings. For instance, there are many potential risks to raisers, members of the public, and puppies, if physically unsuitable raisers are required to handle physically strong and highly distractible or excitable puppies in highly stimulating settings. Given the support available within raisers’ personal networks (e.g., household members, friends, colleagues in the PR program), staff should involve and formally acknowledge the contribution of those individuals. In terms of program design, PR providers should focus their support on ensuring raisers’ awareness of the tasks involved during induction, raisers’ engagement in ongoing effective learning, creating convenient opportunities for raisers to offer their puppy sufficient socialisation experiences, and adhering to program-specific instructions.

### Limitations and Future Directions

This paper devised a behavioural intervention PR model, the 3T model, targeting three practices, with nine specific program functions to guide program providers when developing strategies for individual raisers and organisation wide. This theory-informed model provides organisations with a framework to explain the interconnectedness of PR factors and suggests program outcome criteria for evaluation of their support programs. At this early stage, the 3T model requires collaboration between program providers and researchers interested in behaviour change to evaluate existing support strategies or to develop and implement new strategies consistent with the model. We appreciate that this is a massive and costly undertaking. Therefore, peak bodies, such as the International Guide Dog Federation [32] and Assistance Dogs International [33], should consider supporting research and development in standardised, evidence-based raiser-centred support programs. Finding and retaining effective raisers is a limiting factor for the development of the AD industry. A coordinated approach would improve program experiences for volunteer raisers and thereby improve outcomes for puppies and the overall efficiency of AD providers.

Though we are very confident in directing research attention towards understanding how raiser-related factors determine outcomes for AD puppies, the specifics of the 3T model were developed based on only three recent studies. Since each AD organisation has its own policies and practices, generalisation of the model remains unclear. For instance, some organisations may lack access to stable raiser homes, resulting in puppies having to shift from one raiser to another due to reasons such as raiser unavailability. Puppies sourced from shelters [34] or private breeders may experience disruptions in attachment relationships and living conditions. Such disruptions may put the wellbeing of puppies at risk and also mean that they require extra resources or specific types of care to settle in a new environment before they can benefit from intensive socialisation and training. Only future research will reveal how the 3T model can be adapted for different scenarios. Developing evidence-based, best-practice guidelines would nonetheless be a useful starting point. In addition, because the behaviour change model (i.e., BCW) underlying the 3T model was synthesised from 19 behaviour change theories [16], it is robust. There is a high probability that some effective PR programs have already implemented some of the raiser-centred support features outlined in our 3T model. The framework can therefore help piece together fragmentary evidence, affording a coordinated approach in PR research and practice.

For example, future research may evaluate how raiser learning (i.e., the *Capability* triangle) may benefit from a range of raiser-centred support strategies, including skill training (e.g., modes and intervals of in-person training), skill modelling (e.g., availability of peer-support and mentoring programs) and education (e.g., general canine behaviour, principles of behaviour modelling). In terms of the operationalisation of outcome measures, to date, apart from the self-reported Raiser Practice Questionnaire [14], we are not aware of any measure that focuses on raiser practices. Future research should consider objective measures of raisers’ capability, such as quizzes on their dog training knowledge and direct observations by dog trainers and peers. Comparing raiser-specific outcome measures among groups with and without certain raiser-centred support strategies (i.e., ANOVA or its variations) may prove informative, as may analyses that use more advanced statistical techniques such as multilevel modelling [35]. Importantly, however, when trying to establish the effectiveness of support programs at intrapersonal, interpersonal, and group levels, it is imperative to remain sensitive to the voluntary nature of most PR programs and the vulnerability of AD puppies. Puppies cannot be put at risk by exposure to less-than-optimal practices, and some raisers may perceive any form of assessment as being judgemental of their learning, rather than as a longer-term strategy to improve industry practice. Outcome measures based on the assessment of frequency of socialisation (the *Opportunity* triangle) or consistency of training (i.e., the *Motivation* triangle) may be less difficult to implement, as may existing measures of puppy behaviours. There are many standardised measures of puppy behaviour available (e.g., [4]) so researchers can readily select those most suitable to the intended purposes and age of the puppies.

In short, further understanding of raiser factors contributing to the outcomes of PR programs is imperative for the AD industry. Developing this understanding requires empirical designs and objective measures of various PR factors, which must be supported by a strong theoretical framework, such as that provided by the proposed 3T model. Over time this will inform program providers of more effective operating models, ensuring the efficiency of their investments, positive program experiences for raisers, higher puppy success rates, and more ADs to meet the demands of waiting handlers.

## 5. Conclusions

This paper adopted a systems-level perspective to further understanding of both human and puppy outcomes of assistance dog puppy raising programs. Previous studies reported various factors that influence raiser practices and also demonstrated direct relationships between raiser practices and their ratings of puppy behaviour. Raisers are volunteers who make a long-term commitment to a raising program. Their effectiveness and retention determine the success of the industry, making their subjective program experiences of great interest to AD providers. Integrating a puppy into a raiser’s life and implementing program-specific instructions designed to ensure optimal outcomes may create tension in daily living. Contextual factors such as different sources and types of support influence raiser practices. However, the extent to which raisers benefit from these factors depends on their help-seeking behaviour and the compatibility of available support with their preferred learning modalities. Many factors related to raisers’ daily lives determine their PR practices—which consequently, at least partially, determine whether the puppies they raise are successful. Successful PR may be promoted via a raiser-centred behavioural support approach, but this is dependent on the development of underlying theoretical models and an evidence base. Undertaking this work is imperative because improving raiser practices will benefit puppy behavioural outcomes and, eventually, outcomes for handlers with disabilities who benefit from the important work of assistance dogs.

## Figures and Tables

**Table 1:** Definitions of Behavioural Change Wheel Functions and Puppy Raising Examples.

Descriptions Taken from Michie et al. [16]		Puppy Raising Examples
Interventions	Definition	
Education	Increasing knowledge or understanding	Providing knowledge about puppy socialisation, puppy training and different modalities of ongoing learning for raisers.
Persuasion	Using communication to induce positive or negative feelings or stimulate action	Demonstrating effectiveness of appropriate and sufficient socialisation and training on puppy behaviour.
Incentivisation	Creating expectation of reward	Extending appreciation of raisers’ socialisation and training efforts or including raisers in puppies’ graduation ceremonies.
Coercion	Creating expectation of punishment or cost	Highlighting the financial cost associated with failing a puppy and the cost in terms of added waiting time for potential handlers.
Training	Imparting skills	Imparting analytical skills and specific dog handling techniques.
Restriction	Using rules to reduce the opportunity to engage in the target behaviour (or to increase the target behaviour by reducing the opportunity to engage in competing behaviours)	Limiting access to areas with potential risks (e.g., local parks with potentially aggressive dogs) while promoting access to public places with age-appropriate stimuli.
Environmental restructuring	Changing the physical or social context	Adapting workplace environments so raisers can bring their puppies to work; educating the public to refrain from distracting the puppies.
Modelling	Providing an example for people to aspire to or imitate	Providing opportunities for peer-learning with experienced and competent puppy raisers.
Enablement	Increasing means/reducing barriers to increase capability or opportunity ^1^	Ensuring access to trained puppy sitters who carry out puppy socialisation and training tasks that raisers do not/cannot perform, or in places and at times that are inconvenient to the raisers.

Note: ^1^ Capability beyond education and training; opportunity beyond environmental restructuring [16].

## Data Availability

Not applicable.
